# Proximal Descending Thoracic Aortic Pseudoaneurysm Secondary to Pott's Spine

**DOI:** 10.1055/s-0040-1701212

**Published:** 2020-07-31

**Authors:** Irappa Madabhavi, Malay Sarkar, Chidanand Chauhan, Mitul Modi

**Affiliations:** 1 Department of Medical and Pediatric Oncology and Hematology, Kerudi Cancer Hospital, Bagalkot, Karnataka, India; 2 Department of Pulmonary Medicine, Indira Gandhi Medical College, Shimla, Himachal Pradesh, India; 3 Department of Radiology, Indira Gandhi Medical College, Shimla, Himachal Pradesh, India; 4 Department of Pathology, Gujarat Cancer and Research Institute, Ahmedabad, Gujarat, India

**Keywords:** tuberculosis, vertebra, aneurysm, antitubercular treatment

## Abstract

Tuberculous pseudoaneurysm of the descending thoracic aorta is quite rare, life-threatening, and fatal if not diagnosed in time. This lesion exposes patients to a very high risk of unpredictable rupture. We describe a case of tuberculous pseudoaneurysm of the aorta in association with tuberculosis of the spine (Pott's spine). A 73-year-old man presented with a 2-month history of back pain. Chest roentgenography and contrast-enhanced computed tomography showed a descending thoracic aortic pseudoaneurysm with destruction of the fourth and fifth thoracic vertebrae (T4-T5). We suspected that the pseudoaneurysm was due to direct extension of tuberculous vertebral osteomyelitis. The patient was managed with antituberculous chemotherapy. The post–antitubercular therapy course was uneventful and he remained well 12 months after completion of treatment.

## Introduction

Tubercular pseudoaneurysm of the descending thoracic aorta is a rare and potentially fatal complication of tuberculosis (TB). However, not all cases of aortic involvement by TB lead to aneurysm formation. 1

## Case Presentation

**Fig. 1:**
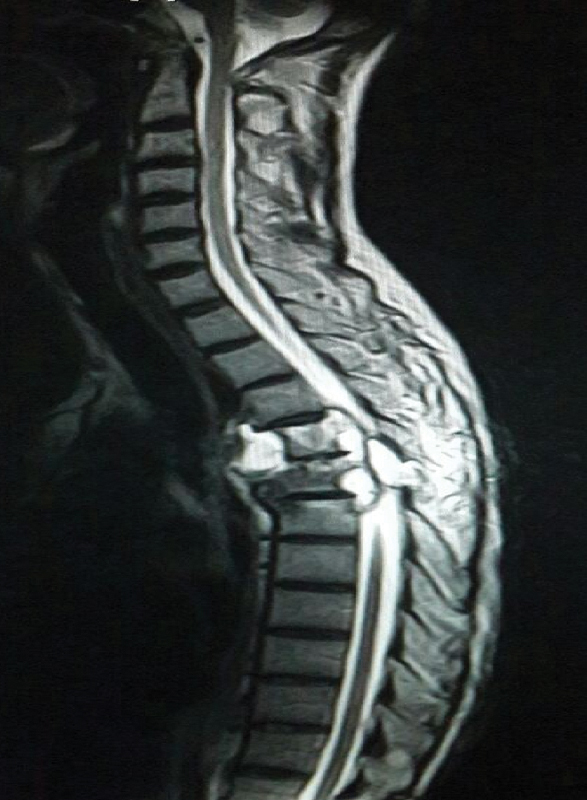
Magnetic resonance imaging showing abnormal signal changes in the T4 and T5 disks and adjacent vertebral bodies with associated prevertebral and epidural soft tissue abnormalities.

**Fig. 2:**
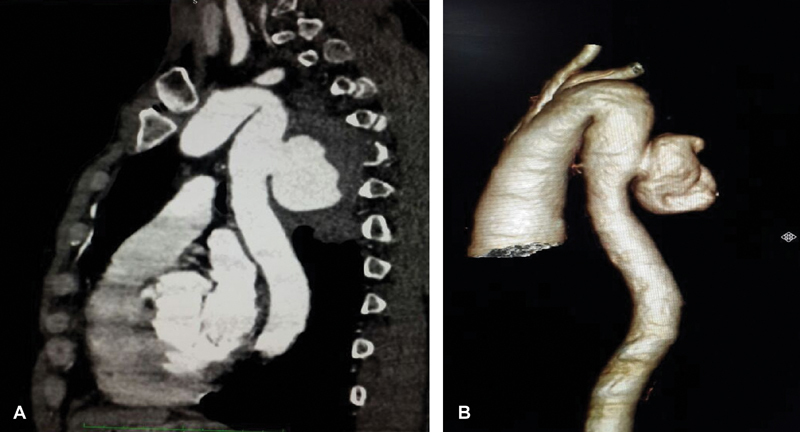
( **A** ) Sagittal and ( **B** ) three-dimensional reconstruction of contrast-enhanced reveals an aortic pseudoaneurysm measuring 52 × 45 × 35 mm involving the posterior wall of the proximal descending thoracic aorta.

A 73-year-old man presented to hospital with a 2-month history of dull back pain. He had no prior history of pulmonary TB. He was not a smoker and had no history of angina, hypertension, dyslipidemia, or ischemic stroke. On admission to hospital, physical examination and laboratory values were normal limits for his age. No causative pathogens were cultured from blood or sputum. Chest roentgenography and magnetic resonance imaging showed a descending thoracic aortic aneurysm with severe destruction of the fourth and fifth thoracic vertebral bodies with pre- and paravertebral soft tissue components ( Fig. 1 ). Contrast-enhanced computed tomographic angiography (CTA) and three-dimensional images reconstruction confirmed a saccular pseudoaneurysm measuring 52 × 45 × 35 mm involving the posterior wall of the proximal descending thoracic aorta with severe destruction of the fourth and fifth thoracic vertebral bodies ( Fig. 2A,B ).

TB aneurysm was confirmed with Mantoux test of more than 15-mm induration, raised erythrocyte sedimentation rate of 80 mm/h, and polymerase chain reaction for *Mycobacterium tuberculosis* deoxyribonucleic acid (DNA) was positive. Since most thoracic aortic aneurysms with corresponding vertebral erosion are associated with mycotic infection, especially TB, we suspected this was a tuberculous aortic aneurysm and initiated antituberculous chemotherapy with isoniazid (0.3 g/d), rifampin (0.45 g/d), ethambutol (0.75 g/d), and pyrazinamide (0.5 g/d) for 9 months.

In view of the old age and poor performance status, after discussion regarding the cardiovascular surgical interventional options, including thoracotomy and cardiopulmonary bypass and also endovascular grafting for this extensive pseudoaneurysm, the patient and the family opted for sole medical management via antitubercular treatment.

We experienced an exceedingly rare case of descending thoracic aortic pseudoaneurysm with severe vertebral erosion at T4 and T5 level, which was likely caused by direct extension of tuberculous vertebral osteomyelitis. Treatment with combined antituberculous chemotherapy was successful, and the patient remains well 12 months after the completion of treatment. After 2-years follow-up, his symptoms and signs disappeared without pseudoaneurysm recurrence by the computed tomography examination.

## Discussion

Tubercular pseudoaneurysm of the descending thoracic aorta is a rare and a potentially fatal complication of TB. However, not all cases of aortic involvement by TB lead to aneurysm formation. 1 There are four mechanisms by which tubercle bacilli may reach the aortic wall: (1) Direct implantation of the bacilli on the internal surface of the aortic wall may occur. Normally, the intima wall is resistant to infection. However, an atherosclerotic patch may increase the risk of direct implantation. Schmorl 2 detected of *tuberculosis bacilli* on atheromatous ulcers in autopsies in 5 of 123 patients with acute miliary TB; (2) The *bacilli* may be carried to the adventitia or media by the vasa vasorum; (3) Involvement of the vessel wall may occur indirectly via the lymphatics 3 ; (4) Direct extension from a neighboring tuberculous lymph node, abscess, or bone may occur. This has been reported in the literature as the most common cause (75%). A contiguous focus of lymph nodes has been described in 63% of the cases, whereas, paraspinal abscess, lung, pericardium, vertebrae, and prostate are the source of infection in 37% cases.

TB mainly involves the thoracic or the abdominal aorta. 4 Involvement of the descending thoracic aorta in association with vertebral TB is rare. Clinically, TB of the aorta remains silent until some major complications develop. The following clinical features of tuberculous aortic aneurysm have been described in the literature. These include persistent chest, abdominal, or back pain, hypovolemic shock or other evidence of major bleeding, particularly into the lung or gastrointestinal tract, but also into the pleural space, peritoneal cavity, retroperitoneum, or pericardial space, and a palpable or radiographically visible para-aortic mass, especially if expanding or pulsatile. 5 Contrast-enhanced computed tomography and magnetic resonance angiography are the modalities commonly used in the detection of tuberculous aortic aneurysm. The characteristic features of tuberculous aortic aneurysm are the absence of pulsation 6 and contrast filling. 7 Other features supporting TB are the presence of a false aneurysm, noninvolvement of the ascending aorta, and radiological presence of a contiguous focus of disease on computed tomography scan. 6 8

Currently, surgery combined with simultaneous anti-TB drug treatment should be used for the disease, especially while considering that no evidence exists to show only anti-TB drug treatment or surgery alone can achieve a cure. Common surgical methods include the following: vascular lesion removal and synthetic vascular replacement; extra-anatomical reconstruction; direct suture closure or patch repair; and endovascular stent-graft exclusion or endovascular aneurysm repair. Currently, the most common surgical approach involves the resection of the diseased segment, removal of the surrounding necrotic tissues, and reconstruction of the distal vessel using a graft from the noninfectious region far from the infected areas (anatomical bypass). 9 10

In summary, we report the case of a 73-year-old male with tuberculous vertebral and pseudoaneurysm formation in the proximal descending thoracic aorta. Pseudoaneurysm is a rare complication of infection by *Mycobacterium tuberculosis* . Contrast-enhanced CTA is the investigation of choice for proper delineation of the aneurysm. Treatment should include a full course of antituberculous chemotherapy and directed vascular surgery. If unrecognized, these aneurysms may rupture, possibly with lethal consequences.
